# Variations in plasma concentrations of tamoxifen metabolites and the effects of genetic polymorphisms on tamoxifen metabolism in Korean patients with breast cancer

**DOI:** 10.18632/oncotarget.22220

**Published:** 2017-11-01

**Authors:** Hye In Woo, Se Kyung Lee, Jiyoung Kim, Seok Won Kim, Jonghan Yu, Soo Youn Bae, Jeong Eon Lee, Seok Jin Nam, Soo-Youn Lee

**Affiliations:** ^1^ Department of Laboratory Medicine, Samsung Changwon Hospital, Sungkyunkwan University School of Medicine, Changwon, Korea; ^2^ Division of Breast Surgery, Department of Surgery, Samsung Medical Center, Sungkyunkwan University School of Medicine, Seoul, Korea; ^3^ Department of Surgery, Jeju National University School of Medicine, Jeju National University Hospital, Jeju, Korea; ^4^ Division of Breast and Endocrine Surgery, Department of Surgery, Korea University Anam Hospital, Korea University College of Medicine, Seoul, Korea; ^5^ Department of Clinical Pharmacology & Therapeutics, Samsung Medical Center, Seoul, Korea; ^6^ Department of Laboratory Medicine & Genetics, Samsung Medical Center, Sungkyunkwan University School of Medicine, Seoul, Korea

**Keywords:** tamoxifen, metabolite, drug monitoring, genotype, variation

## Abstract

Inter-individual variation in tamoxifen metabolism in breast cancer patients is caused by various genetic and clinical factors. We measured the plasma concentrations of tamoxifen and its metabolites and investigated genetic polymorphisms influencing those concentrations. We measured the concentrations of tamoxifen, endoxifen, N-desmethyltamoxifen (NDM), and 4-hydroxytamoxifen (4-OH tamoxifen) in 550 plasma specimens from 281 breast cancer patients treated with tamoxifen. Duplicate or triplicate specimens were obtained from 179 patients at 3-month intervals. In 80 patients, genotyping for tamoxifen metabolizing enzymes was performed using the DMET Plus array and long-range PCR. Plasma concentrations of tamoxifen and its metabolites showed wide variations among patients. The following genetic polymorphisms were associated with the plasma concentrations when body mass index and tamoxifen concentrations were considered as co-variables: *CYP1A2* -2467delT, *CYP2B6* genotype, CYP2D6 activity score (AS), and *FMO3* 441C>T. CYP2D6 AS and three variants in the *SULT1E1* gene showed correlation with ratios of tamoxifen metabolites. CYP2D6 AS was the only variable that showed associations with both metabolite concentration and ratio: endoxifen (*P* < 0.001), NDM (*P* < 0.001), endoxifen/NDM (*P* < 0.001), NDM/tamoxifen (*P* < 0.001), and 4-OH tamoxifen/tamoxifen (*P* = 0.005). Serial measurements of 448 plasma concentrations in 179 patients at 3-month intervals showed wide intra-individual variation. Our study showed that genetic polymorphisms can in part determine the baseline concentrations of tamoxifen and its metabolites. However, marked intra-individual variations during follow-up monitoring were observed, and this could not be explained by genotype. Therefore, serial measurements of tamoxifen and its metabolites would be helpful in monitoring *in vivo* tamoxifen metabolic status.

## INTRODUCTION

Tamoxifen is an antiestrogenic drug widely used in the treatment of estrogen receptor (ER)-and/or progesterone receptor (PR)-positive breast cancers [1]. Five year treatment with adjuvant tamoxifen leads to around a 30%-50% reduction in the recurrence rate throughout the first 10 years and a 30% reduction in mortality rates throughout the first 15 years in early ER-positive breast cancer [2, 3]. A recent large study reported that 10 year tamoxifen treatment reduced the recurrence rate and mortality by 15%-30% more than stopping tamoxifen after 5 years [4]. The benefit of continuing tamoxifen treatment longer than 10 years following surgery is not clear; thus, adjuvant tamoxifen treatment is recommended for up to 10 years [5]. Clinical outcomes of treatment with tamoxifen show inter-individual variation, which can be caused by various factors including concomitant medication and genetic polymorphisms of enzymes involved in the metabolic pathway of tamoxifen [2, 6–8].

**Figure 1:**
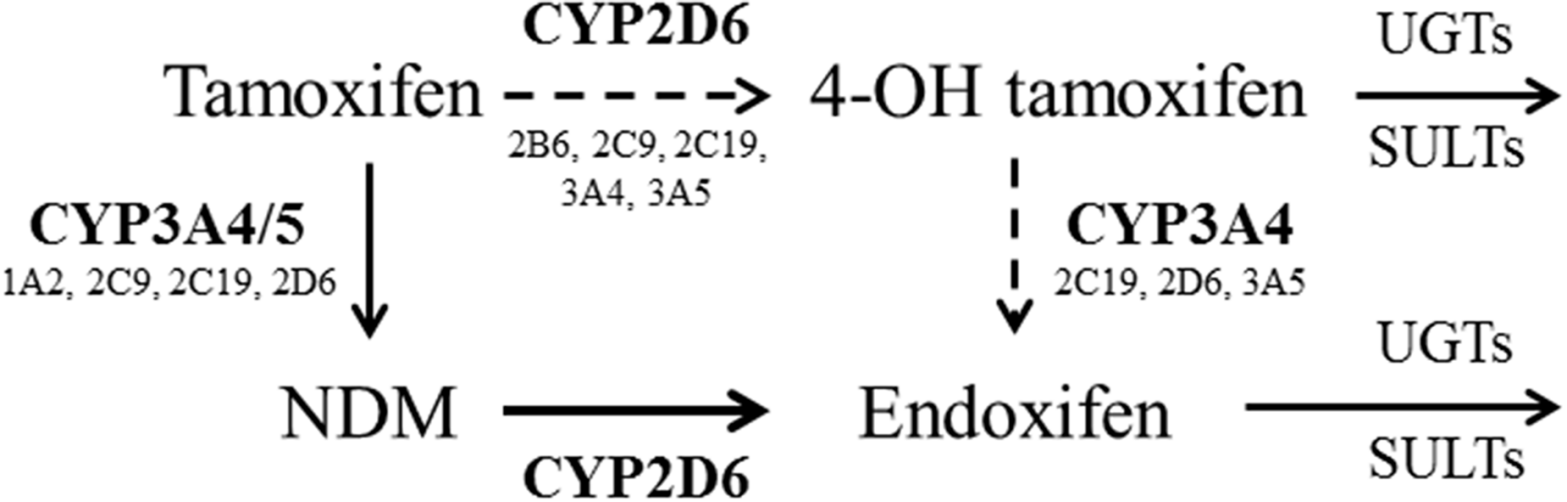
The metabolic pathway of tamoxifen Tamoxifen is demethylated to N desmethyltamoxifen (NDM) mainly by CYP3A4/5. A relatively smaller proportion of tamoxifen is converted by CYP2D6 to 4-hydroxytamoxifen (4-OH tamoxifen). NDM is metabolized to endoxifen by CYP2D6, and 4-OH tamoxifen is metabolized to endoxifen by CYP3A4 [11]. In addition to these enzymes, various CYP enzymes (e.g., CYP1A2, CYP2C9, etc.) are involved in the metabolism of tamoxifen. Tamoxifen and its metabolites are further inactivated by UDP-glucuronosyltransferases (UGTs) and sulfotransferases (SULTs) [14, 15].

Tamoxifen and its metabolites are ER antagonists that competitively inhibit estrogen binding to ERs [9]. In addition, some of them are also known to inhibit aromatase [10]. Tamoxifen undergoes a complex metabolic process involving the cytochrome P450 (CYP) system in the liver [11]. N-desmethyltamoxifen (NDM) generated from demethylation of tamoxifen by CYP3A4/5 is a major primary metabolite of tamoxifen (Figure 1) [11]. A relatively smaller proportion of tamoxifen is converted by CYP2D6 to 4-hydroxytamoxifen (4-OH tamoxifen), which is an active metabolite with strong affinity for ERs and 30- to 100-fold higher potency in suppressing estrogen-dependent cell proliferation [2, 11]. Other minor primary metabolites of tamoxifen, including alpha-hydroxytamoxifen, have also been identified [11]. 4-hydroxy-N-desmethyltamoxifen (endoxifen) is biotransformed from NDM and 4-OH tamoxifen by CYP2D6 and CYP3A [11]. Endoxifen has potent antiestrogenic activity (100-fold more potent than tamoxifen) and shows higher plasma concentrations than 4-OH tamoxifen [2, 11–13]. In addition to endoxifen, NDM is also metabolized to secondary metabolites, including N-didesmethyltamoxifen and alpha-hydroxy-NDM, by various CYP enzymes [11]. Tamoxifen and its metabolites are further inactivated by UDP-glucuronosyltransferases (UGTs) and sulfotransferases (SULTs) [14, 15]. In addition to these enzymes, various CYP and flavin-containing monooxygenase (FMO) enzymes are also involved in tamoxifen metabolism [11, 16].

Variant alleles of *CYP2D6* genes have been reported to be associated with clinical outcomes, including recurrence rates, mortality rates, and disease-free survival, in breast cancer patients treated with tamoxifen; these include functional alleles related to better clinical outcomes and non-functional or reduced function alleles associated with worse outcomes [17]. In contrast, some studies have reported the absence of clear evidence for associations between genetic polymorphisms of drug-metabolizing enzymes and clinical outcomes [18, 19]. These inconsistencies among pharmacogenetic studies were suggested to be due to various metabolic pathways and various potent metabolites of tamoxifen [20]. *In vivo* studies on the impact of genotype on tamoxifen pharmacokinetics revealed that *CYP2D6* genotypes are associated with different concentrations of tamoxifen metabolites, mainly endoxifen, in a patient-dependent manner [2, 21–26]. In addition to *CYP2D6*, the influence of various genes, including *CYP3A4*, *CYP2C9*, *CYP2C19*, *SULT1A1*, *SULT1A2*, and *UGT*s, on tamoxifen metabolism has been occasionally reported [27–34]. However, genetic polymorphisms appear to only explain part of the inter-individual variation of tamoxifen metabolism, and most previous studies focused on a few polymorphisms in a small proportion of genes in tamoxifen metabolism [2, 8, 30]. Moreover, there are still insufficient data on genetic diversity with respect to geographic background and ethnicity. In addition to genetic factors, clinical factors including concomitant medicine and compliance also contribute to the variation in tamoxifen metabolism. These factors can cause fluctuation in the tamoxifen metabolic state in each patient during long-term treatment. However, the intra-individual variation in the tamoxifen metabolic state in breast cancer patients has barely been evaluated.

The aims of this study were to determine concentrations of tamoxifen and its metabolites in plasma specimens, including serial specimens collected at 3-month intervals, from Korean breast cancer patients and to investigate the impacts of genetic polymorphisms in multiple genes on tamoxifen pharmacokinetics. The results of this study will improve our understanding of the effects of genetic polymorphisms on tamoxifen metabolism and inter- and intra-individual variations in the concentrations of tamoxifen metabolites.

## RESULTS

### Patient characteristics

Table 1 summarizes the demographics and clinical characteristics of 281 breast cancer patients treated with tamoxifen. The mean age of the patients was 45.4 years (range, 27.3-73.7 years). At the time of blood draw, mean body mass index (BMI) was 22.9 kg/m^2^ (16.8-36.4 kg/m^2^), and the mean duration of tamoxifen treatment before blood collection was 125 days (56-340 days). Most patients had normal liver function; the mean values of AST and ALT were 22 IU/L (5-86 IU/L) and 18 IU/L (5-118 IU/L), respectively. Most patients (74.0%) were in a pre/peri-menopausal state. Three patients reported taking CYP2D6 inhibitors including escitalopram, hydroxyzine, and ranitidine [35]. Except one who was taking hydroxyzine before the third blood collection time, the other two patients discontinued CYP2D6 inhibitors at least 2 weeks prior to the second blood collection time.

**Table 1:** Clinical characteristics of 281 patients with breast cancer

Characteristics	Mean (range) or n (%)
Age (years)	45.4 (27.3-73.7)
Body mass index (kg/m^2^)	22.9 (16.8-36.4)
Menopausal status, n (%)	
Pre/peri-menopause	208 (74.0)
Post-menopause	73 (26.0)
Tamoxifen duration at initial blood collection time (days)	125 (56-340)
Dose of tamoxifen (mg/day)	20
Neoadjuvant chemotherapy	
No	274 (97.5)
Yes	7 (2.5)
Adjuvant chemotherapy	
No	148 (52.7)
Yes	133 (47.3)
Stage	
0	61 (21.7)
1	118 (42.0)
2	74 (26.3)
3	28 (10.0)
Nuclear grade	
1	78 (27.8)
2	132 (47.0)
3	68 (24.2)
Unknown	3 (1.1)
Number of measurement, n (%)	
Once	102 (36.3)
Twice	89 (31.7)
Thrice	90 (32.0)
Concomitant medicine, n (%)^a^	
Goserelin	43 (7.8)
Levothyroxin	11 (2.0)
Calcium carbonate/cholecalciferol	7 (1.3)
Valsaltan	6 (1.1)
Amlodipine	5 (0.9)
CYP inhibitor^b^	3 (0.5)
Others^c^	25 (4.5)
Not used	473 (86.0)

^a^Denominator is the total number of blood collection time (550).

^b^Escitalopram, hydroxyzine, and ranitidine.

^c^Aceclofenac, atorvastatin, alendronate, calcium polycarbophil, cefdinir, cefditoren, gabapentin, gliclazide, herceptin, hydrochlorothiazide, insulin, itopride, lamotrigine, losartan/hydrochlothiazide, magnesium oxide, metformin, pethidine, ramipril, risedronate, rosuvastatin, sitagliptin, tizanidine, tobramycin, tramadol, triazolam, valaciclovir, venlafaxine, voglibose, zaltoprofen, zoledronic acid, and zolpidem.

### Plasma concentrations of tamoxifen and its metabolites

**Figure 2:**
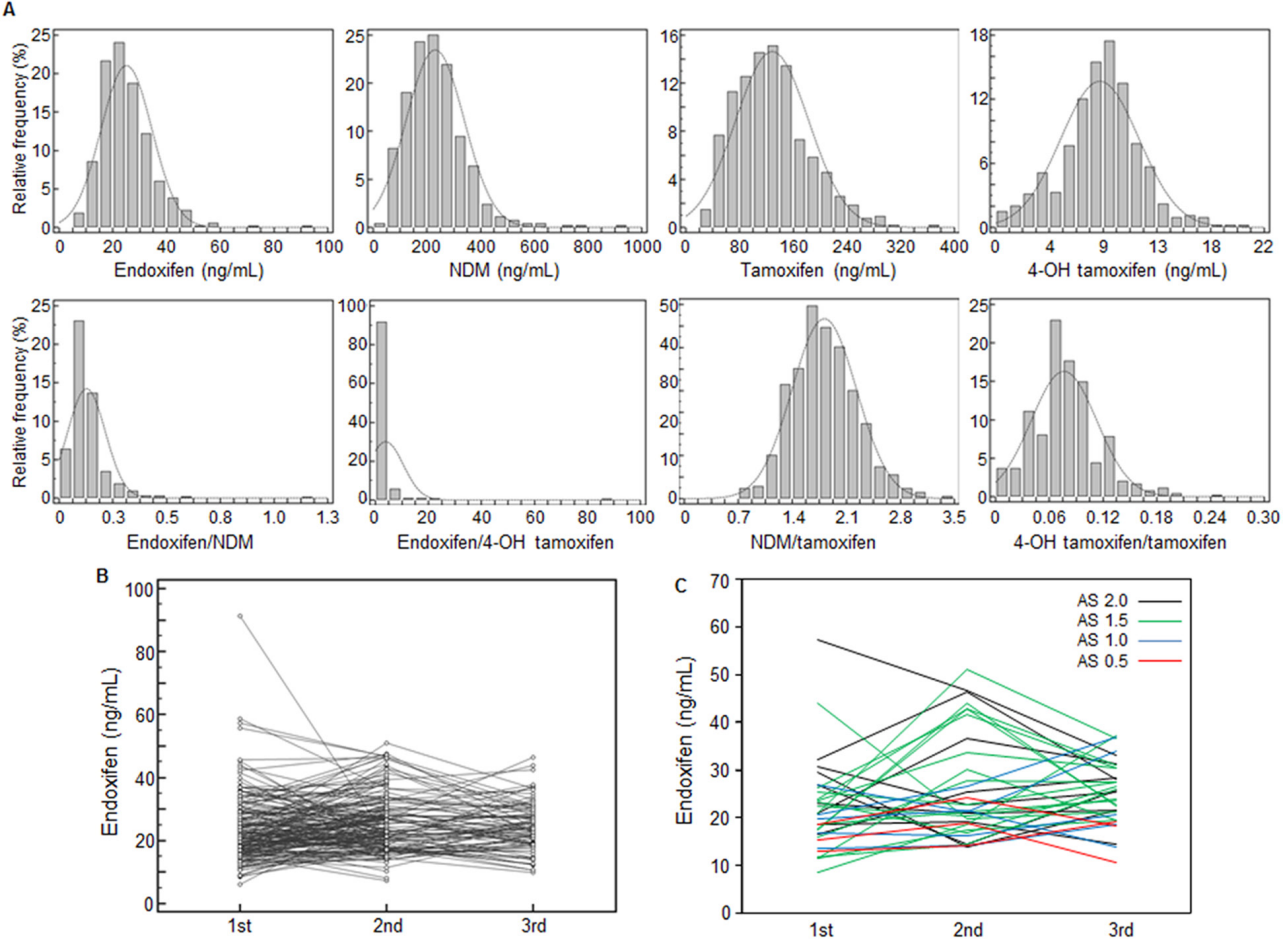
Distribution of and changes in the concentrations of tamoxifen and its metabolites **(A)** Histogram of tamoxifen metabolite concentrations (ng/mL) and ratios in 550 plasma specimens. Tamoxifen metabolites and ratios showed wide variation (CV, 30-274%). **(B)** Plasma concentrations of endoxifen at 3-month intervals in 179 patients; 0.23- to 4.87-fold changes were observed in endoxifen concentrations over the 3-month interval. **(C)** Plasma concentrations of endoxifen according to CYP2D6 AS in 33 patients. Endoxifen concentrations at 3-month intervals showed wide variations regardless of the CYP2D6 AS. AS, activity score; NDM, N-desmethyltamoxifen; 4-OH tamoxifen, 4-hydroxytamoxifen.

A total of 550 measurements of tamoxifen and its metabolites were obtained for 281 patients taking 20 mg daily tamoxifen (Table 2). Among the three tamoxifen metabolites, NDM (230 ng/mL) had the highest mean concentration compared with endoxifen (25.0 ng/mL) and 4-OH tamoxifen (8.53 ng/mL). The concentrations of tamoxifen metabolites and their ratios were widely distributed (Figure 2A). The ratio of endoxifen and 4-OH tamoxifen showed the highest variation, with a coefficient of variation (CV) of 274%. For the active metabolites endoxifen and 4-OH tamoxifen, the fifth percentile of concentration showed an approximate 3-fold difference from the 95th percentile (12.5 vs. 42.7 ng/mL for endoxifen, 2.60 vs. 13.4 ng/mL for 4-OH tamoxifen). In comparisons of concentrations and clinical variables, BMI showed statistical significance with endoxifen and tamoxifen, but the degree of correlation was very weak (*r*^2^ = 0.018, *P* = 0.002 for endoxifen; *r*^2^ = 0.011, *P* = 0.014 for tamoxifen). Concentrations of tamoxifen and its metabolites measured at 3-month intervals were inconsistent; plasma endoxifen concentration changed 0.23- to 4.87-fold (Figure 2B), and 21 cases (7.8%, 21/269) had a greater than 2-fold change; among these cases, the endoxifen concentration was decreased at 3 months in seven cases. One patient who was taking a CYP2D6 inhibitor, hydroxyzine, showed a decrease in endoxifen concentration from 26.8 ng/mL before to 18.5 ng/mL after hydroxyzine administration. Another patient was non-compliant and showed a fluctuation in endoxifen concentration from 29.6 ng/mL at first blood collection, 13.9 ng/mL at the second, to 21.5 ng/mL at the third.

**Table 2:** The distributions of concentrations of tamoxifen and its metabolites in 550 plasma specimens of 281 patients

Variables	Concentrations (95% CI) in ng/mL							
	Endoxifen	NDM	Tam	4-OH Tam	Endoxifen /NDM	Endoxifen /4-OH Tam	NDM /Tam	4-OH Tam /Tam
Mean	25.0 (24.2-25.8)	230 (221-240)	128 (124-133)	8.53 (8.26-8.80)	0.13 (0.12-0.14)	4.27 (3.29-5.25)	1.83 (1.78-1.87)	0.08 (0.07-0.08)
SD	9.47	108	54.5	3.21	0.09	11.7	0.54	0.04
CV	38%	47%	43%	38%	69%	274%	30%	50%
5th percentile	12.5 (11.4-13.6)	80.5 (71.5-92.8)	50.3 (44.6-56.0)	2.60 (2.00-3.40)	0.05 (0.05-0.05)	1.82 (1.74-1.86)	1.18 (1.12-1.24)	0.02 (0.01-0.02)
25th percentile	18.5 (17.7-19.4)	157 (145-168)	89.4 (83.5-95.2)	6.70 (6.20-7.20)	0.08 (0.08-0.08)	2.37 (2.30-2.45)	1.51 (1.48-1.58)	0.05 (0.05-0.06)
50th percentile	23.4 (22.5-24.4)	223 (215-231)	123 (117-129)	8.75 (8.50-9.00)	0.11 (0.10-0.11)	2.82 (2.76-2.87)	1.79 (1.74-1.82)	0.08 (0.07-0.08)
75th percentile	30.2 (29.1-31.2)	282 (271-298)	158 (153-165)	10.4 (10.1-10.7)	0.15 (0.14-0.17)	3.45 (3.33-3.62)	2.08 (2.03-2.14)	0.10 (0.09-0.10)
95th percentile	42.7 (40.1-44.8)	413 (380-438)	231 (218-245)	13.4 (12.9-14.4)	0.29 (0.26-0.33)	7.18 (5.40-9.05)	2.53 (2.45-2.69)	0.14 (0.13-0.15)

CI, confidence interval; CV, coefficient of variation; NDM, N-desmethyltamoxifen; 4-OH Tam, 4-hydroxytamoxifen; SD, standard deviation; Tam, tamoxifen.

### Genotype frequencies

Genotype frequencies are summarized in Table 3 and Table 4. For the *CYP2D6* gene, the CYP2D6^*^10 allele was identified in 42.5% of all alleles, and *CYP2D6* ^*^10/^*^10 homozygotes accounted for 17.5% of all patients. In the analysis according to the activity score (AS) predicted by *CYP2D6* genotype, compared to the homozygote of the functional allele (2 AS, 23.8%), 46.3% of patients showed lower activity of 1.5 AS, followed by those with 1 AS (25.0%). For the *CYP2B6* gene, the CYP2B6^*^6 allele associated with decreased enzyme activity was most frequently identified (12.9%). The allele frequencies of CYP2C9^*^3, CYP2C19^*^2, and CYP2C19^*^3 were 4.2%, 29.3%, and 5.7% respectively. The frequency of the CYP3A5^*^3 allele (74.3%) was higher than that of the wild-type allele. Regarding the *SULT1A1* and *UGT2B7* genes, the ^*^2 variant allele was most frequent (7.6% and 23.2%, respectively). For the *CYP1A2*, *FMO1*, *FMO3*, *NR1I2*, *SULT1E1*, and *UGT2B15* genes, genotype frequencies of each variant are presented in Table 4. The *CYP1A2* -2467delT variant was most frequent (47.5%). *SULT1E1* -9-469A>G and *SULT1E1* -10+311G>C showed complete linkage disequilibrium.

**Table 3:** Genotype frequencies of *CYP2B6, CYP2C9, CYP2C19, CYP2D6, CYP3A4, CYP3A5, SULT1A1, SULT2A1, *and *UGT2B7* genes

Gene	Genotype	Predicted phenotype	n	%
*CYP2B6*	^*^1/^*^1	NF/NF	34	54.8
	^*^1/^*^2B	NF/NF	2	3.2
	^*^1/^*^4	NF/IOF	7	11.3
	^*^1/^*^5	NF/NF	1	1.6
	^*^1/^*^6	NF/DOF	12	19.4
	^*^1/^*^7	NF/NF	2	3.2
	^*^1/^*^22	NF/IOF	1	1.6
	^*^2B/^*^6	NF/DOF	2	3.2
	^*^6/^*^6	DOF/DOF	1	1.6
*CYP2C9*	^*^1/^*^1	NF/NF	64	88.9
	^*^1/^*^3	NF/DOF	6	8.3
	^*^1/^*^7	NF/NF	2	2.8
*CYP2C19*	^*^1/^*^1	NF/NF	25	35.7
	^*^1/^*^2	NF/LOF	29	41.4
	^*^1/^*^3	NF/LOF	3	4.3
	^*^1/^*^17	NF/IOF	4	5.7
	^*^2/^*^2	LOF/LOF	4	5.7
	^*^2/^*^3	LOF/LOF	3	4.3
	^*^2/^*^17	LOF/IOF	1	1.4
	^*^3/^*^3	LOF/LOF	1	1.4
*CYP2D6*	^*^1/^*^1	NF/NF (2)^a^	12	15.0
	^*^1/^*^2	NF/NF (2)	7	8.8
	^*^1/^*^5	NF/LOF (1)	4	5.0
	^*^1/^*^10	NF/DOF (1.5)	22	27.5
	^*^1/^*^41	NF/DOF (1.5)	3	3.8
	^*^2/^*^10	NF/DOF (1.5)	8	10.0
	^*^5/^*^10	LOF/DOF (0.5)	3	3.8
	^*^6/^*^10	LOF/DOF (0.5)	1	1.3
	^*^10/^*^10	DOF/DOF (1)	14	17.5
	^*^10/^*^10xN	DOF/DOF, xN (1.5)	1	1.3
	^*^10/^*^39	DOF/NF (1.5)	3	3.8
	^*^10/^*^41	DOF/DOF (1)	2	2.5
*CYP3A4*	^*^1/^*^1	Wt/Wt	78	97.5
	^*^1/^*^5	Wt/Vt^b^	1	1.3
	^*^1/^*^18	Wt/Vt	1	1.3
*CYP3A5*	^*^1/^*^1	NF/NF	5	3.4
	^*^1/^*^3	NF/DOF	27	18.2
	^*^3/^*^3	DOF/DOF	41	27.7
	^*^3/^*^5	DOF/DOF	1	0.7
*SULT1A1*	^*^1/^*^1	Wt/Wt	67	84.8
	^*^1/^*^2	Wt/Vt	12	15.2
*SULT2A1*	^*^1/^*^1	Wt/Wt	80	100.0
*UGT2B7*	^*^1/^*^1	Wt/Wt	28	40.6
	^*^1/^*^2	Wt/Vt	27	39.1
	^*^1/^*^3	Wt/Vt	8	11.6
	^*^2/^*^3	Vt/Vt	5	7.2
	^*^3/^*^3	Vt/Vt	1	1.4

DOF, decrease of function; IOF, increase of function; LOF, loss of function; NF, normal function; Vt, variant; Wt, wild type.

^a^Activity scores of *CYP2D6* gene in parentheses.

^b^For genes whose phenotype is not well predicted by the genotype, the presence or absence of the variant allele is presented.

**Table 4:** Genotype frequencies of *CYP1A2, FMO1, FMO3, NR1I2, SULT1E1, *and*UGT2B15* genes

Polymorphism	Genotype, n (%)		
	Wild homozygote	Heterozygote	Variant homozygote
*CYP1A2* -3860G>A	46 (57.5)	30 (37.5)	4 (5.0)
*CYP1A2* -2467delT	21 (26.3)	42 (52.5)	17 (21.3)
*CYP1A2* -739T>G	70 (87.5)	10 (12.5)	0 (0.0)
*CYP1A2* -163A>C	32 (41.6)	40 (51.9)	5 (6.5)
*CYP1A2* 5347C>T	52 (65.0)	26 (32.5)	2 (2.5)
*FMO1* 747C>T	78 (97.5)	2 (2.5)	0 (0.0)
*FMO1* 1188A>G	78 (97.5)	2 (2.5)	0 (0.0)
*FMO1* ^*^111C>T	43 (54.4)	31 (39.2)	5 (6.3)
*FMO1* ^*^207C>T	41 (51.3)	34 (42.5)	5 (6.3)
*FMO3* 441C>T	48 (60.0)	30 (37.5)	2 (2.5)
*FMO3* 472G>A	55 (68.8)	22 (27.5)	3 (3.8)
*FMO3* 627+10C>G	78 (97.5)	2 (2.5)	0 (0.0)
*FMO3* 769G>A	58 (75.3)	15 (19.5)	4 (5.2)
*FMO3* 923A>G	56 (70.9)	19 (24.1)	4 (5.1)
*NR1I2* -1135C>T	41 (52.6)	31 (39.7)	6 (7.7)
*SULT1E1* -64G>A	40 (50.0)	32 (40.0)	8 (10.0)
*SULT1E1* -9-899G>A	48 (60.0)	27 (33.8)	5 (6.3)
*SULT1E1* -9-682A>G	67 (84.8)	12 (15.2)	0 (0.0)
*SULT1E1* -9-469A>G	34 (42.5)	35 (43.8)	11 (13.8)
*SULT1E1* -10+311G>C	34 (42.5)	35 (43.8)	11 (13.8)
*UGT2B15* 253G>T	18 (22.5)	46 (57.5)	16 (20.0)
*UGT2B15* 1568A>C	63 (78.8)	14 (17.5)	3 (3.8)
*UGT2B15* ^*^185A>T	46 (57.5)	29 (36.3)	5 (6.3)

Genotype frequencies of variants with minor allele frequency ≥ 1% and call rate > 85% are listed.

### Associations between genotypes and plasma concentrations of tamoxifen and its metabolites

**Figure 3:**
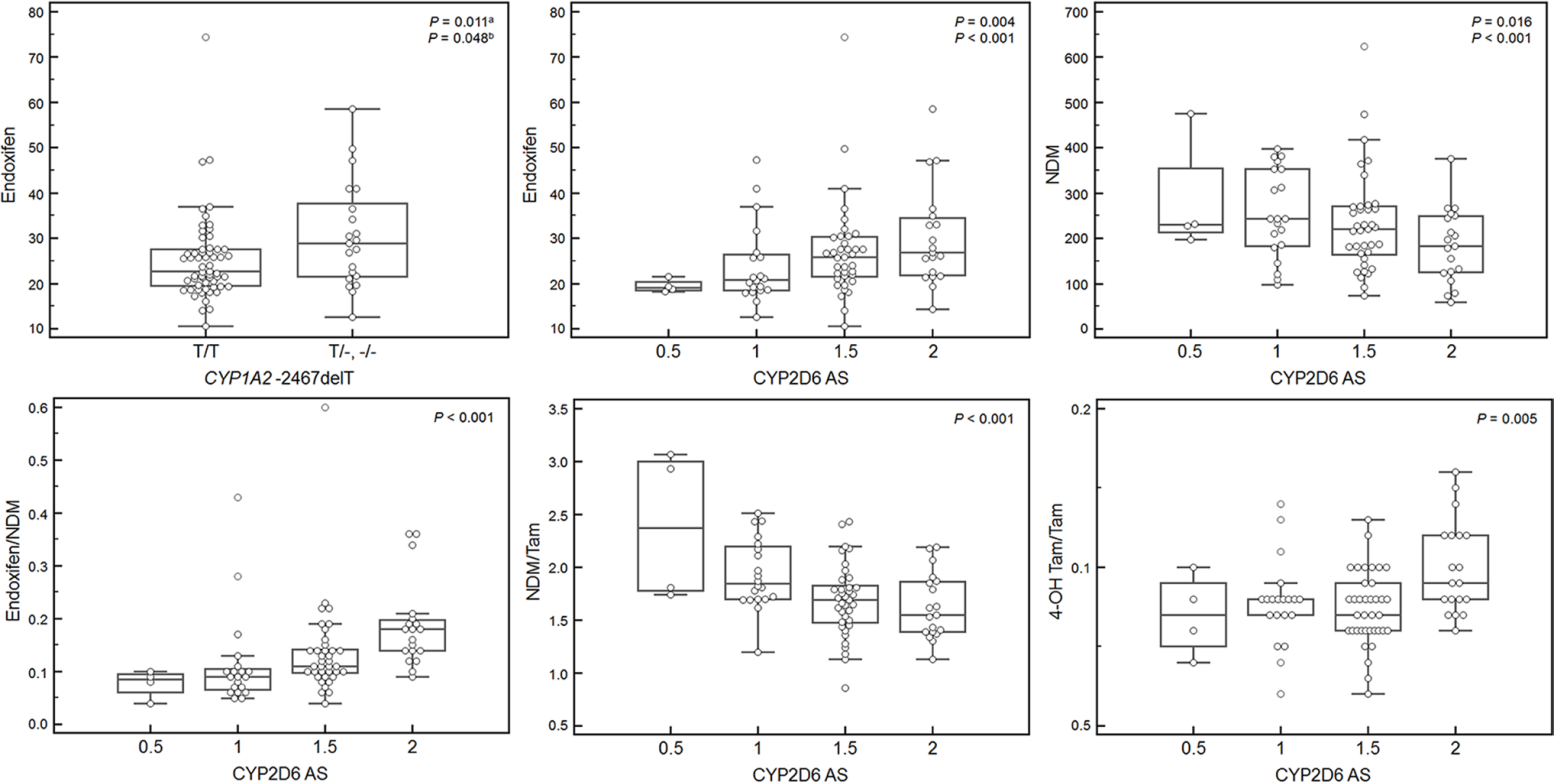
Concentrations of plasma tamoxifen metabolites and their ratios according to *CYP1A2* -2467delT genotype and *CYP2D6* activity score (AS) Box-plots show differences in endoxifen concentration according to classification based on the genotype of *CYP1A2* -2467delT and differences in the concentrations of tamoxifen metabolites and their ratios according to CYP2D6 AS among associations with multivariable *P*-value less than 0.050 in Table 5. The other statistical significances between metabolite concentrations and genotypes or predicted phenotypes are presented in Table 5. Deletion of nucleotide T in *CYP1A2* c.-2467 resulted in higher endoxifen concentrations than wild type. The concentrations and ratios of endoxifen, endoxifen/NDM, and 4-OH tamoxifen/tamoxifen were positively correlated with CYP2D6 AS, and NDM and NDM/tamoxifen were negatively correlated with CYP2D6 AS. NDM, N-desmethyltamoxifen; 4-OH tamoxifen, 4-hydroxytamoxifen; Tam, tamoxifen.

^a^Multivariable *P*-value including genetic variants with univariate *P*-values < 0.100 and body mass index.

^b^*P*-value from multivariable analysis including tamoxifen concentration as an indicator of compliance and drug absorption.

Various genotypes showed associations with tamoxifen and its metabolites in univariate analysis. For genotypes showing a statistically significant association with the concentrations of tamoxifen and its metabolites in multivariable analysis, i.e., *CYP1A2* -2467delT, *CYP2B6* genotype, CYP2D6 AS, *FMO3* 441C>T, *SULT1E1* -9-899G>A, *SULT1E1* -9-682A>G, *SULT1E1* -9-469A>G, and *UGT2B7* genotype, the mean plasma concentrations according to genotype and predicted phenotype are presented in Table 5. The 4-OH tamoxifen/tamoxifen ratio increased with BMI (*P* = 0.024). In multivariable analysis including tamoxifen and BMI in addition to variables with univariate *P*-values < 0.100, concentrations of metabolites showed correlations with *CYP1A2* -2467delT, *CYP2B6* genotype, *CYP2D6* AS, and *FMO3* 441C>T. The mean concentration of endoxifen (*P* < 0.001) was decreased and that of NDM (*P* < 0.001) was increased in accord with the decrease in CYP2D6 AS (Figure 3). The ratios of tamoxifen and its metabolites, endoxifen/NDM, NDM/tamoxifen, and 4-OH tamoxifen/tamoxifen, also showed associations with CYP2D6 AS. Patients who were heterozygous or homozygous for *CYP1A2* -2467delT had lower endoxifen concentrations than those who were homozygous wild-type (25.3 vs. 30.5 ng/mL, *P* = 0.048). *FMO3* 441C>T was associated with high NDM concentration (206 ng/mL for CC genotype vs. 265 ng/mL for CT and TT genotypes, *P* = 0.027). For 4-OH tamoxifen, the concentration tended to be increased in accord with a decrease in CYP2B6 activity (*P* = 0.002). 4-OH tamoxifen was found to be decreased according to the increase in the number of variants of the *UGT2B7* gene, but statistical significance was not conserved when considering tamoxifen concentration (*P* = 0.122). In addition to CYP2D6 AS, three variants on the *SULT1E1* gene, *SULT1E1* -9-899G>A, *SULT1E1* -9-682A>G, and *SULT1E1* -9-469A>G, affected the ratio of tamoxifen and its metabolites. There was no genotype effect on the ratio of endoxifen and 4-OH tamoxifen. Figure 2C shows multiple endoxifen measurements classified by the CYP2D6 AS; patients showed a wide variation of endoxifen concentrations regardless of the CYP2D6 AS.

**Table 5:** Mean concentrations of tamoxifen and its metabolites according to genotypes and predicted phenotypes

Variables (n)	Mean concentrations (95% CI) in ng/mL							
	Endoxifen	NDM	Tam	4-OH Tam	Endoxifen /NDM	Endoxifen /4-OH Tam	NDM /Tam	4-OH Tam /Tam
*CYP1A2* -2467delT								
TT (21)	30.5 (25.2-35.8)	243 (209-276)	149 (125-172)	10.3 (8.78-11.9)	0.13 (0.11-0.15)	3.03 (2.64-3.42)	1.68 (1.54-1.82)	0.07 (0.06-0.08)
T- + -- (59)	25.3 (22.8-27.8)	225 (195-254)	123 (110-137)	9.27 (8.64-9.90)	0.14 (0.12-0.17)	2.83 (2.56-3.11)	1.80 (1.69-1.91)	0.08 (0.08-0.09)
*P*	0.043^a^ (0.011)^b^ (0.048)^c^	0.503	0.062 (0.108)	0.125	0.899	0.303	0.216	0.066 (0.314)
*CYP2B6* genotype								
NF/IOF (8)	20.5 (15.0-26.1)	216 (162-270)	123 (87.0-158)	7.20 (4.45-9.95)	0.10 (0.06-0.14)	3.44 (1.91-4.96)	1.79 (1.62-1.95)	0.06 (0.03-0.09)
NF/NF (52)	27.2 (24.3-30.0)	238 (207-268)	133 (120-146)	9.69 (9.06-10.3)	0.14 (0.11-0.17)	2.86 (2.59-3.12)	1.77 (1.65-1.89)	0.08 (0.07-0.08)
NF/DOF (14)	30.1 (22.9-37.2)	244 (185-302)	146 (104-187)	11.0 (8.93-13.0)	0.14 (0.10-0.18)	2.76 (2.31-3.21)	1.76 (1.57-1.94)	0.09 (0.07-0.10)
DOF/DOF (1)	27.6	265	109	9.80	0.10	2.82	2.43	0.09
*P*	0.031 (0.077) (0.487)	0.539	0.429	0.005 (0.004) (0.002)	0.335	0.361	0.654	0.039 (0.628)
*CYP2D6* AS								
2 (19)	30.1 (24.8-35.4)	184 (145-223)	116 (87.8-144)	10.2 (8.75-11.6)	0.19 (0.15-0.23)	2.94 (2.66-3.22)	1.63 (1.48-1.78)	0.10 (0.09-0.11)
1.5 (37)	27.1 (23.5-30.7)	234 (197-270)	138 (120-155)	9.54 (8.59-10.5)	0.14 (0.11-0.17)	2.95 (2.60-3.30)	1.68 (1.57-1.80)	0.07 (0.07-0.08)
1 (20)	24.0 (19.9-28.2)	254 (209-300)	132 (109-155)	9.30 (8.22-10.4)	0.11 (0.07-0.16)	2.73 (2.15-3.32)	1.93 (1.78-2.09)	0.08 (0.07-0.08)
0.5 (4)	19.5 (17.2-21.7)	283 (77.3-489)	119 (65.9-171)	7.90 (3.72-12.1)	0.08 (0.04-0.12)	2.78 (0.75-4.81)	2.39 (1.26-3.52)	0.07 (0.03-0.11)
*P*	0.009 (0.004) (< 0.001)	0.018 (0.012) (< 0.001)	0.581	0.132	< 0.001 (< 0.001)	0.181	< 0.001 (< 0.001)	0.010 (0.005)
*FMO3* 441C>T								
CC (48)	27.0 (23.4-30.6)	206 (182-230)	122 (107-136)	9.26 (8.39-10.1)	0.15 (0.12-0.19)	3.04 (2.69-3.39)	1.73 (1.61-1.84)	0.08 (0.07-0.09)
CT + TT (32)	26.2 (23.8-28.6)	265 (221-309)	143 (123-163)	9.99 (9.17-10.8)	0.12 (0.10-0.14)	2.65 (2.45-2.86)	1.83 (1.70-1.97)	0.08 (0.07-0.08)
*P*	0.802	0.013 (0.002) (0.027)	0.076 (0.139)	0.242	0.063 (0.134)	0.141	0.237	0.298
*SULT1E1* -9-899G>A								
GG (48)	26.6 (23.8-29.5)	252 (219-285)	139 (122-156)	9.76 (8.89-10.6)	0.12 (0.11-0.14)	2.83 (2.55-3.11)	1.83 (1.72-1.95)	0.08 (0.07-0.08)
GA (27)	26.9 (22.2-31.6)	200 (172-228)	120 (107-133)	9.28 (8.32-10.2)	0.16 (0.11-0.21)	3.00 (2.54-3.47)	1.67 (1.51-1.83)	0.08 (0.07-0.09)
AA (5)	25.4 (13.9-36.9)	171 (15.1-326)	99.4 (12.4-186)	8.96 (6.72-11.2)	0.19 (0.06-0.32)	2.79 (2.04-3.55)	1.70 (1.33-2.08)	0.11 (0.06-0.17)
*P*	0.881	0.014 (0.005) (0.190)	0.045 (0.014)	0.383	0.022 (0.180)	0.636	0.117	0.022 (0.006)
*SULT1E1* -9-899G>A								
GG (48)	26.6 (23.8-29.5)	252 (219-285)	139 (122-156)	9.76 (8.89-10.6)	0.12 (0.11-0.14)	2.83 (2.55-3.11)	1.83 (1.72-1.95)	0.08 (0.07-0.08)
GA+AA (32)	26.7 (22.6-30.8)	195 (167-224)	117 (102-131)	9.23 (8.40-10.1)	0.16 (0.12-0.21)	2.97 (2.57-3.37)	1.67 (1.53-1.81)	0.09 (0.08-0.10)
*P*	0.967	0.015	0.068	0.399	0.038	0.530	0.076 (0.049)	0.141
*SULT1E1* -9-682A>G								
AA (67)	27.3 (24.7-30.0)	234 (208-259)	130 (117-143)	9.58 (8.91-10.3)	0.14 (0.12-0.16)	2.94 (2.68-3.20)	1.81 (1.71-1.91)	0.08 (0.07-0.09)
AG + GG (12)	23.1 (19.4-26.8)	209 (140-277)	131 (89.4-172)	9.35 (7.50-11.2)	0.14 (0.09-0.18)	2.60 (2.08-3.12)	1.59 (1.46-1.71)	0.08 (0.06-0.10)
*P*	0.199	0.444	0.971	0.784	0.915	0.216	0.078 (0.017)	0.841
*SULT1E1* -9-469A>G								
AA+AG (69)	26.8 (24.2-29.3)	221 (196-247)	127 (114-139)	9.44 (8.76-10.1)	0.15 (0.12-0.17)	2.94 (2.69-3.20)	1.75 (1.65-1.84)	0.08 (0.08-0.09)
GG (11)	25.9 (20.4-31.5)	281 (227-334)	152 (120-183)	10.2 (9.03-11.4)	0.10 (0.07-0.13)	2.51 (2.19-2.84)	1.90 (1.65-2.15)	0.07 (0.06-0.08)
*P*	0.950	0.077 (0.451) (0.251)	0.148	0.380	0.059 (0.019)	0.195	0.220	0.245
*UGT2B7* genotype								
^*^1/^*^1 (28)	28.4 (23.7-33.0)	231 (180-282)	135 (112-158)	10.2 (9.07-11.4)	0.15 (0.12-0.19)	2.73 (2.47-2.99)	1.69 (1.50-1.88)	0.08 (0.07-0.09)
^*^1/^*^2, ^*^1/^*^3 (35)	26.4 (22.8-30.0)	236 (210-262)	132 (118-146)	9.02 (8.07-9.98)	0.13 (0.09-0.16)	3.15 (2.70-3.60)	1.81 (1.70-1.92)	0.07 (0.06-0.08)
^*^2/^*^3, ^*^3/^*^3 (6)	20.5 (13.7-27.4)	158 (92.7-224)	80.7 (53.7-108)	8.38 (7.36-9.41)	0.16 (0.05-0.27)	2.44 (1.79-3.09)	1.93 (1.52-2.33)	0.11 (0.08-0.15)
*P*	0.145	0.329	0.080 (0.186)	0.057 (0.020) (0.122)	0.583	0.818	0.135	0.429

AS, activity score; BMI, body mass index; CI, confidence interval; NDM, N-desmethyltamoxifen; 4-OH Tam, 4-hydroxytamoxifen; Tam, tamoxifen.

^a^Univariable p-value.

^b^Multivariable *p*-value.

^c^*P*-value from multivariable analysis including tamoxifen concentration as indicator for compliance and drug absorption.

## DISCUSSION

In this study, we measured the plasma concentrations of tamoxifen and its metabolites in breast cancer patients treated with tamoxifen and confirmed significant variation among patients. We also evaluated the effects of genetic polymorphisms of multiple genes and clinical factors on tamoxifen metabolism.

The plasma concentrations of tamoxifen and its metabolites showed inter-individual variation among patients receiving the same dose of tamoxifen. To identify clinical and genetic factors for the prediction of these inter-individual variations and to expand the range of pharmacokinetics predicted by genetic factors, we included multiple genetic polymorphisms of tamoxifen metabolizing enzymes. Based on our data, we observed an association between *CYP1A2* -2467delT, *CYP2B6* genotype, CYP2D6 AS, *FMO3* 441C>T, *SULT1E1* -9-899G>A, *SULT1E1* -9-682A>G, *SULT1E1* -9-469A>G, and *UGT2B7* genotype and the plasma concentrations of tamoxifen and its metabolites. When we included tamoxifen concentration as a compliance indicator in our multivariable analysis of metabolite concentrations, the statistical significances of *CYP1A2* -2467delT and CYP2D6 AS for endoxifen, CYP2D6 AS and *FMO3* 441C>T for NDM, and the *CYP2B6* genotype for 4-OH tamoxifen were conserved. CYP2D6 AS showed the most statistically significant effects on various metabolites and ratios. CYP2D6 is known to be the main enzyme responsible for the metabolism of tamoxifen, leading to variability in the concentrations of endoxifen and 4-OH tamoxifen [2, 24]. The CYP1A2 enzyme is known to affect only a small part of the metabolism of NDM to endoxifen [11]. Our study also showed that *CYP1A2* -2467delT correlated with endoxifen concentration, but not with the ratio of metabolites, which suggests a minor contribution of CYP1A2 to tamoxifen metabolism and the possibility of other contributing factors. Three polymorphisms of the *SULT1E1* gene were associated with the ratio of tamoxifen metabolites, suggesting that the concentration of primary metabolites of tamoxifen can be influenced by enzymes located in a distant metabolic pathway. However, these three polymorphisms have not been evaluated in previous studies. Although previous studies have reported the roles of the *SULT1A1* and *SULT1A2* genes in tamoxifen metabolism [27, 28], the association between tamoxifen metabolite concentration and the *SULT1E1* gene, which is involved in sulfation of 4-OH tamoxifen to 4-OH tamoxifen sulfate, has barely been studied clinically [28]. *SULT1E1* -64G>A (rs3736599) homozygotes showed low endoxifen concentrations [28], but the difference was not statistically significant as our study (mean endoxifen concentration in our study, 27.0 ng/mL in wild homozygotes and heterozygotes vs. 23.7 ng/mdL in variant homozygotes, *P* = 0.389). UGT2B7^*^2 (rs7439366) was reported to be correlated with concentrations of tamoxifen and its metabolites in some [33, 34], but not other studies [8, 30]. When we simultaneously analyzed UGT2B7^*^2 and ^*^3, low metabolite concentrations were observed in patients with a variant allele, but statistical significance was not conserved after multivariate correction including tamoxifen concentration. The difference observed with respect to *UGT2B7* variant in our study seemed to have been caused by differences in compliance and absorption. BMI was correlated with the 4-OH tamoxifen/tamoxifen ratio in our study. In previous studies, age was correlated with the concentrations of tamoxifen, endoxifen, and NDM [36], and BMI and ethnicity were also reported to be related with endoxifen concentration [24]. In our study, although several variants showed correlations with biochemical phenotypes as described above, there was no variant that correlated with both metabolic concentration and corresponding ratio except CYP2D6 AS and *SULT1E1* -9-899G>A. We evaluated multiple genetic polymorphisms and various clinical variables as candidates to explain the inter-individual variation in tamoxifen metabolism. Nevertheless, these factors only partially explained the inter-individual variability of *in vivo* metabolite concentrations (adjusted R square less than 0.300 for each metabolite and their ratios). Previous studies also reported that *CYP2D6* genotypes could only explain 39% and 9%, respectively, of the variability of the concentrations of endoxifen and 4-OH tamoxifen [2, 30]. Although *CYP2D6* only explains a part of the variability in metabolic concentration, our findings suggest a low possibility of other genetic variants or combinations that could explain the variation of tamoxifen metabolism better than CYP2D6.

Measurements from each patient at 3-month intervals showed remarkable intra-individual variation in our study, despite the fact that the time required to reach steady state had already passed in those patients. A previous study reported that variations in the intra-patient concentrations of tamoxifen and endoxifen were small at two sampling points with at least a 4-week interval [37]. Our study monitored patients at 3-months intervals given the long-term tamoxifen use. Additionally, the large intra-individual variation in this condition well reflects the routine clinical setting. Even when we classified the serial measurements according to CYP2D6 AS, most patients showed fluctuations in plasma endoxifen concentration regardless of CYP2D6 AS group. This finding shows that genetic polymorphisms have limitations in being able to account for the changes in concentration of tamoxifen and its metabolites *in vivo*, and tamoxifen metabolism would also be influenced by alterations in the clinical status of each patient. However, except in four patients who were either non-compliant or used CYP inhibitors, we could not identify any specific factor that might influence tamoxifen and its metabolite concentrations. This reflects the difficulty in determining variable factors in a routine clinical setting. Therefore, although genetic polymorphisms might be able to help physicians decide on the initial dose of tamoxifen, it would be necessary to monitor factors reflecting the current status of each patient. In many cases, clinical and environmental factors that cause alterations of the tamoxifen metabolic status cannot be identified; thus, monitoring using direct measurements of plasma tamoxifen and its metabolites would be more informative for estimating tamoxifen dosing and other confounding factors including compliance and concomitant medications. Recently, a studies on tamoxifen dose escalation based on endoxifen concentration was published [38, 39]. Although more evidence is required to confirm the correlation between endoxifen concentration and clinical outcome, this study showed that the best method for determining endoxifen exposure is to measure endoxifen concentration rather than to evaluate clinical and genetic factors and indicated the possibility of tamoxifen dose adjustment using therapeutic drug monitoring [38, 39].

A limitation of our study was that rare genotypes such as *CYP2D6* ^*^5/^*^6 were not evaluated. Although there is no universal consensus for translating *CYP2D6* genotypes into phenotypic categories, previous studies have shown that the concentrations of tamoxifen and its metabolites differ according to CYP2D6 phenotype predicted by genotype [24, 30, 40]. Considering the differences in plasma concentrations associated with CYP2D6 AS even among CYP2D6 extensive metabolizers in our study, we suggest that the *CYP2D6* gene has a potent relationship with tamoxifen metabolism. Another limitation is that genotyping was performed in only approximately one-third of patients, which limits the power of statistical analysis. Previous studies showed a correlation between endoxifen concentration and recurrence or relapse-free survival of breast cancer [24, 41]. However, the association between drug concentration and clinical outcome has not been validated; thus, validation through further studies will be required. The strength of our study is that we addressed the genotype frequencies of various genes on tamoxifen metabolism in a Korean population and showed intra-individual variation of tamoxifen metabolic status even in the same genotype-predicted phenotype group. The frequency of functional alleles is different among each ethnic population, with around 71% of Caucasians and 50% of Asians having functional alleles [7]; thus, the correlation between variants and clinical and biochemical phenotypes will need to be validated in each ethnic group.

In this study, we showed that various polymorphisms in tamoxifen metabolizing enzymes account in part for inter-individual variations in the plasma concentrations of tamoxifen and its metabolites. Among the multiple genes evaluated, we confirmed a prominent role of the *CYP2D6* gene as a genetic factor affecting tamoxifen metabolism. However, tamoxifen metabolism could not be predicted by genetic polymorphisms alone, as we observed by serial measurements of plasma tamoxifen and its metabolites. Our results showed intra-individual variations even in patients belonging to the same CYP2D6 phenotype group. In this respect, our study suggests that therapeutic drug monitoring of plasma tamoxifen and its metabolites would be helpful in evaluating patient compliance, insufficient tamoxifen dose, various unknown confounding factors that are involved in tamoxifen metabolism, and in establishing an individualized therapeutic plan.

## MATERIALS AND METHODS

### Patients

This study included patients who received adjuvant tamoxifen. Two hundred eighty-one Korean patients who were pathologically diagnosed with ER+ and/or PR+ breast cancer at Samsung Medical Center were retrospectively included. Patients were treated with 20 mg daily tamoxifen with or without ovarian suppression using the GnRH agonist goserelin after surgery, which included either breast-conserving surgery followed by adjuvant radiotherapy or mastectomy. The study excluded patients with the following conditions: metastatic disease at diagnosis, pregnancy, breast feeding, active tuberculosis, acute myocardial infarction within 6 months, uncontrolled angina pectoris, heart failure, forced expiratory volume in one second (FEV1) less than 60% of that of a healthy population, serum creatinine ≥ 2.0 mg/dL, serum total bilirubin ≥ 2.0 mg/dL, history of aspirin administration within 7 days or anticoagulants, and concomitant CYP2D6 inhibitors at initial tamoxifen administration. Compliance was evaluated during a routine regulatory visit by counting the remaining number of doses. Plasma concentrations of tamoxifen and its metabolites were measured at least 8 weeks after initiating tamoxifen therapy. Among these patients, to measure the plasma concentrations of tamoxifen and its metabolites, a second blood sample was collected 3 months after the initial blood sampling in 182 patients, and a third blood sample was collected in 88 patients. Peripheral blood specimens were collected before taking tamoxifen. Clinical and laboratory data at the time of blood collection including age, sex, BMI, liver function test, menopausal state, and concomitant medications was collected. Menopause was defined as amenorrhea of greater than 12 consecutive months. This study was approved by the Samsung Medical Center Institutional Review Board. Informed consent was obtained from all patients.

### Metabolite analysis

Concentrations of tamoxifen, 4-OH tamoxifen, NDM, and endoxifen were measured in a total of 550 plasma specimens. Plasma concentrations of tamoxifen and its metabolites were measured by high-performance liquid chromatography-tandem mass spectrometry [42, 43]. Analyses were performed on an API 4000 tandem mass spectrometer (AB Sciex, Foster City, CA, USA) equipped with an Agilent Technologies Series 1200 HPLC system (Agilent Technologies, Santa Clara, CA, USA). The column used was a Poroshell 120 EC-C18 (2.7 μm, 3 mm × 50 mm). The mobile phases A and B were water with 2 mM ammonium acetate and acetonitrile, respectively, both containing 0.1% formic acid. For simple protein precipitation, the plasma samples were mixed with acetonitrile containing an internal standard (IS, diphenhydramine) and centrifuged for 5 min. Quantitative analysis was performed in multiple reaction-monitoring mode (m/z 372.2 → 72.2 for tamoxifen, 388.2 → 72.3 for 4-OH tamoxifen, 358.2 → 58.2 for NDM, 374.3 → 58.1 for endoxifen, and 256.2 → 167.0 for IS) with a total running time of 5 min for each sample. Intra- and inter-day coefficients of variation were lower than 10%.

### Genotyping

Tests for genetic variation in tamoxifen-metabolizing enzymes were performed in 80 of a total of 281 patients. Genomic DNA was extracted from peripheral blood leukocytes using the Wizard® Genomic DNA Purification Kit according to the manufacturer’s instructions (Promega, Fitchburg, WI, USA). Extracted DNA was stored at -70°C. Genotyping of drug metabolizing enzymes was performed using the DMET Plus array (Affymetrix, Santa Clara, CA, USA) according to the protocol described in the user guide. The arrays were scanned with a GeneChip® Scanner 3000 7G (Affymetrix), and genotype calls were generated with DMET Console software (Affymetrix). Long-range PCR was performed to identify common deletions or duplications in the *CYP2D6* gene [44]. In total, 211 polymorphisms in 16 genes in the tamoxifen metabolic pathway (*CYP1A2*, *CYP2B6*, *CYP2C9*, *CYP2C19*, *CYP2D6*, *CYP3A4*, *CYP3A5*, *FMO1*, *FMO3*, *NR1I2*, *SULT1A1*, *SULT1E1*, *SULT2A1*, *UGT1A4*, *UGT2B7*, and *UGT2B15*) were genotyped. One polymorphism was excluded due to a Hardy–Weinberg equilibrium (HWE) p-value less than 0.001. Finally, 210 polymorphisms in 16 genes were included in this study (Supplementary Table 1). Genotype was assigned according to the combination of previously known variants for *CYP2B6*, *CYP2C9*, *CYP2C19*, *CYP2D6*, *CYP3A4*, *CYP3A5*, *SULT1A1*, *SULT2A1*, and *UGT2B7* genes.

### Statistical analysis

Plasma concentrations of tamoxifen, 4-OH tamoxifen, NDM, and endoxifen and their ratios were tested for normal distribution. Variables without a normal distribution (endoxifen, the ratio of endoxifen/NDM, and the ratio of endoxifen/4-OH tamoxifen) were converted to a normal distribution by applying a logarithmic scale before statistical analyses. Comparisons of plasma concentrations of tamoxifen, 4-OH tamoxifen, NDM, endoxifen, and their ratios with clinical variables and genotypes were performed by simple regression analysis. Variants with call rates higher than 85% were included in the statistical analysis and in assignment of genotype and predicted phenotype. For genes with previously known genotypes and a predicted phenotype (*CYP2B6*, *CYP2C9*, *CYP2C19*, *CYP3A4*, *CYP3A5*, *SULT1A1*, *SULT2A1*, and *UGT2B7*), the plasma concentrations of tamoxifen and its metabolites and their ratios were compared with the predicted phenotype or the presence or absence of the variant allele (Table 3). For *CYP2D6*, patients were classified according to AS based on previous studies and statistically analyzed [45–48]. For genes whose phenotypes were not well predicted by their genotypes (*CYP1A2*, *FMO1*, *FMO3*, *NR1I2*, *SULT1E1*, and *UGT2B15*), variants with minor allele frequency greater than or equal to 1% were evaluated according to additive, dominant, and recessive genetic models. Genetic variants and clinical variables with univariate *P*-value < 0.100 and BMI were included as adjusted variables for the multiple regression analysis. Multiple variable analysis including tamoxifen concentration was also performed for metabolite concentrations to evaluate drug absorption and compliance. A *P*-value < 0.050 was regarded to be statistically significant. Statistical analyses were performed using IBM SPSS Statistics version 20.0 (SPSS Inc., Chicago, IL, USA).

## SUPPLEMENTARY MATERIALS TABLE





## Abbreviations

AS: activity score
BMI: body mass index
CV: coefficient of variation
CYP: cytochrome P450
ER: estrogen receptor
FMO: flavin-containing monooxygenase
4-OH tamoxifen: 4-hydroxytamoxifen
NDM: N-desmethyltamoxifen
PR: progesterone receptor
SULTs: sulfotransferases
UGTs: UDP-glucuronosyltransferases
