# Supplementary material for: Variations in plasma concentrations of tamoxifen metabolites and the effects of genetic polymorphisms on tamoxifen metabolism in Korean patients with breast cancer
Source: Oncotarget. 2017 Nov 1;8(59):100296–311. doi: 10.18632/oncotarget.22220 (PMC5725021; doi:10.18632/oncotarget.22220)
Supplement: Supplementary file 1 [file oncotarget-08-100296-s001.pdf]

Variations in plasma concentrations of tamoxifen metabolites and the effects of genetic polymorphisms on tamoxifen metabolism in Korean patients with breast cancer

SUPPLEMENTARY MATERIALS

Supplementary Table 1: Summary of 210 polymorphisms in 16 genes encoding tamoxifen-metabolizing enzymes

See Supplementary File 1
