# Supplementary material for: Variations in plasma concentrations of tamoxifen metabolites and the effects of genetic polymorphisms on tamoxifen metabolism in Korean patients with breast cancer
Source: Oncotarget. 2017 Nov 1;8(59):100296–311. doi: 10.18632/oncotarget.22220 (PMC5725021; doi:10.18632/oncotarget.22220)
Supplement: Supplementary file 2 [file oncotarget-08-100296-s002.doc]

**Supplementary Table 1:** Summary of 210 polymorphisms in 16 genes encoding tamoxifen-metabolizing enzymes.

| **Gene** | **Polymorphisms** | **rs no.** | **Call rate (%)** | **MAF (%)** |
| --- | --- | --- | --- | --- |
| *CYP1A2* | CYP1A2 -3860G>A | rs2069514 | 100 | 23.4 |
| *CYP1A2* | CYP1A2 -2467delT | rs35694136 | 97.8 | 46.1 |
| *CYP1A2* | CYP1A2 -739T>G | rs2069526 | 100 | 5.4 |
| *CYP1A2* | CYP1A2 -729C>T | rs12720461 | 94.6 | 0.0 |
| *CYP1A2* | CYP1A2 -163C>A | rs762551 | 95.7 | 34.1 |
| *CYP1A2* | CYP1A2 63C>G | rs56160784 | 100 | 0.0 |
| *CYP1A2* | CYP1A2 125C>G | rs72547511 | 95.7 | 0.0 |
| *CYP1A2* | CYP1A2 558C>T | rs72547513 | 100 | 0.0 |
| *CYP1A2* | CYP1A2 2116G>A | rs56276455 | 100 | 0.0 |
| *CYP1A2* | CYP1A2 2385G>A | rs72547514 | 100 | 0.0 |
| *CYP1A2* | CYP1A2 2473G>A | rs72547515 | 100 | 0.0 |
| *CYP1A2* | CYP1A2 2499A>T | rs72547516 | 97.8 | 0.0 |
| *CYP1A2* | CYP1A2 3496G>A | rs55889066 | 100 | 0.0 |
| *CYP1A2* | CYP1A2 3533G>A | rs56107638 | 66.3 | 0.0 |
| *CYP1A2* | CYP1A2 5090C>T | rs28399424 | 100 | 0.0 |
| *CYP1A2* | CYP1A2 5166G>A | rs72547517 | 100 | 0.0 |
| *CYP1A2* | CYP1A2 5347C>T | rs2470890 | 98.9 | 18.1 |
| *CYP2B6* | CYP2B6 -82T>C | rs34223104 | 100 | 0.5 |
| *CYP2B6* | CYP2B6 64C>T | rs8192709 | 90.2 | 3.0 |
| *CYP2B6* | CYP2B6 136A>G | rs35303484 | 88.0 | 0.0 |
| *CYP2B6* | CYP2B6 12740G>C | rs2279341 | 88.0 | 3.1 |
| *CYP2B6* | CYP2B6 12820G>A | rs36060847 | 100 | 0.0 |
| *CYP2B6* | CYP2B6 13072A>G | rs12721655 | 100 | 0.0 |
| *CYP2B6* | CYP2B6 13076G>A | rs35773040 | 100 | 0.0 |
| *CYP2B6* | CYP2B6 14593C>G | rs4803418 | 75.0 | 44.9 |
| *CYP2B6* | CYP2B6 15582C>T | rs4803419 | 98.9 | 48.4 |
| *CYP2B6* | CYP2B6 15614C>G | rs3826711 | 98.9 | 0.0 |
| *CYP2B6* | CYP2B6 15618C>T | rs36056539 | 100 | 0.0 |
| *CYP2B6* | CYP2B6 15631G>T | rs3745274 | 100 | 15.2 |
| *CYP2B6* | CYP2B6 15708T>C | rs36079186 | 60.9 | 0.0 |
| *CYP2B6* | CYP2B6 18045C>A | rs45482602 | 95.7 | 0.0 |
| *CYP2B6* | CYP2B6 18053A>G | rs2279343 | 98.9 | 18.7 |
| *CYP2B6* | CYP2B6 18273G>A | rs2279344 | 94.6 | 31.0 |
| *CYP2B6* | CYP2B6 21011T>C | rs28399499 | 75.0 | 0.0 |
| *CYP2B6* | CYP2B6 21034C>T | rs34826503 | 98.9 | 0.0 |
| *CYP2B6* | CYP2B6 21160C>T | rs34097093 | 100 | 0.0 |
| *CYP2B6* | CYP2B6 21388T>A | rs35979566 | 100 | 0.0 |
| *CYP2B6* | CYP2B6 21498C>A | rs35010098 | 100 | 0.0 |
| *CYP2B6* | CYP2B6 21563C>T | rs8192719 | 100 | 15.2 |
| *CYP2B6* | CYP2B6 25505C>T | rs3211371 | 67.4 | 2.4 |
| *CYP2C19* | CYP2C19 -806C>T | rs12248560 | 100 | 3.3 |
| *CYP2C19* | CYP2C19 1A>G | rs28399504 | 100 | 0.0 |
| *CYP2C19* | CYP2C19 50T>C | rs55752064 | 100 | 0.0 |
| *CYP2C19* | CYP2C19 55A>C | rs17882687 | 100 | 0.0 |
| *CYP2C19* | CYP2C19 12460G>A | rs17878459 | 98.9 | 0.0 |
| *CYP2C19* | CYP2C19 12711T>C | rs41291556 | 100 | 0.0 |
| *CYP2C19* | CYP2C19 12784G>A | rs17884712 | 100 | 0.0 |
| *CYP2C19* | CYP2C19 17948G>A | rs4986893 | 89.1 | 6.1 |
| *CYP2C19* | CYP2C19 19153C>T | rs6413438 | 100 | 0.0 |
| *CYP2C19* | CYP2C19 19154G>A | rs4244285 | 98.9 | 25.8 |
| *CYP2C19* | CYP2C19 19194_19195insG | rs72558185 | 100 | 0.0 |
| *CYP2C19* | CYP2C19 19294T>A | rs72558186 | 100 | 0.0 |
| *CYP2C19* | CYP2C19 80161G>A | rs3758581 | 100 | 5.4 |
| *CYP2C19* | CYP2C19 87290C>T | rs17879685 | 100 | 0.0 |
| *CYP2C19* | CYP2C19 90209A>C | rs55640102 | 100 | 0.0 |
| *CYP2C19* | CYP2C19 90033C>T | rs56337013 | 100 | 0.0 |
| *CYP2C9* | CYP2C9 55C>A | rs67807361 | 90.2 | 1.8 |
| *CYP2C9* | CYP2C9 269T>C | rs72558187 | 100 | 0.0 |
| *CYP2C9* | CYP2C9 374G>A | rs72558189 | 100 | 0.0 |
| *CYP2C9* | CYP2C9 430C>T | rs1799853 | 100 | 0.0 |
| *CYP2C9* | CYP2C9 485C>A | rs72558190 | 100 | 0.0 |
| *CYP2C9* | CYP2C9 752A>G | rs2256871 | 100 | 0.0 |
| *CYP2C9* | CYP2C9 815A>G | rs9332130 | 98.9 | 0.0 |
| *CYP2C9* | CYP2C9 818delA | rs9332131 | 100 | 0.0 |
| *CYP2C9* | CYP2C9 895A>G | rs72558192 | 100 | 0.0 |
| *CYP2C9* | CYP2C9 1003C>T | rs28371685 | 98.9 | 0.0 |
| *CYP2C9* | CYP2C9 1073A>G | rs1057909 | 100 | 0.0 |
| *CYP2C9* | CYP2C9 1075A>C | rs1057910 | 100 | 4.3 |
| *CYP2C9* | CYP2C9 1076T>C | rs56165452 | 100 | 0.0 |
| *CYP2C9* | CYP2C9 1080C>G | rs28371686 | 100 | 0.0 |
| *CYP2C9* | CYP2C9 1323C>T | rs2017319 | 85.9 | 0.0 |
| *CYP2C9* | CYP2C9 1425A>T | rs1057911 | 87.0 | 1.9 |
| *CYP2C9* | CYP2C9 1465C>T | rs9332239 | 100 | 0.0 |
| *CYP2D6* | CYP2D6 -1770G>A | rs1080983 | 100 | 12.5 |
| *CYP2D6* | CYP2D6 -1584C>G | rs1080985 | 100 | 9.8 |
| *CYP2D6* | CYP2D6 100C>T | rs1065852 | 93.5 | 47.7 |
| *CYP2D6* | CYP2D6 124G>A | rs5030862 | 81.5 | 0.0 |
| *CYP2D6* | CYP2D6 1023C>T | rs28371706 | 65.2 | 0.0 |
| *CYP2D6* | CYP2D6 1659G>A | rs61736512 | 97.8 | 0.0 |
| *CYP2D6* | CYP2D6 1661G>C | rs1058164 | 92.4 | 35.3 |
| *CYP2D6* | CYP2D6 1707delT | rs5030655 | 82.6 | 1.3 |
| *CYP2D6* | CYP2D6 1758G>A | rs5030865 | 76.1 | 0.0 |
| *CYP2D6* | CYP2D6 1846G>A | rs3892097 | 85.9 | 0.6 |
| *CYP2D6* | CYP2D6 1863_1864insTTTCGCCCC | rs72549356 | 100 | 0.0 |
| *CYP2D6* | CYP2D6 1973_1974insG | rs72549354 | 96.7 | 0.0 |
| *CYP2D6* | CYP2D6 2539_2542delAACT | rs72549353 | 77.2 | 0.0 |
| *CYP2D6* | CYP2D6 2549delA | rs35742686 | 100 | 0.0 |
| *CYP2D6* | CYP2D6 2573_2574insC | rs72549352 | 100 | 0.0 |
| *CYP2D6* | CYP2D6 2587_2590delGACT | rs72549351 | 89.1 | 0.0 |
| *CYP2D6* | CYP2D6 2850C>T | rs16947 | 100 | 12.0 |
| *CYP2D6* | CYP2D6 2935A>C | rs5030867 | 97.8 | 0.0 |
| *CYP2D6* | CYP2D6 2950G>C | rs72549349 | 100 | 0.0 |
| *CYP2D6* | CYP2D6 2988G>A | rs28371725 | 100 | 2.7 |
| *CYP2D6* | CYP2D6 3183G>A | rs59421388 | 75 | 0.0 |
| *CYP2D6* | CYP2D6 3259_3260insGT | rs72549346 | 98.9 | 0.0 |
| *CYP2D6* | CYP2D6 4180G>C | rs1135840 | 83.7 | 35.7 |
| *CYP3A4* | CYP3A4 -392A>G | rs2740574 | 100 | 0.0 |
| *CYP3A4* | CYP3A4 44T>C | rs12721634 | 100 | 0.0 |
| *CYP3A4* | CYP3A4 6004G>A | rs56324128 | 100 | 0.0 |
| *CYP3A4* | CYP3A4 11451A>G | rs3091339 | 100 | 0.0 |
| *CYP3A4* | CYP3A4 13871A>G | rs55951658 | 100 | 0.0 |
| *CYP3A4* | CYP3A4 13908G>A | rs72552799 | 96.7 | 0.0 |
| *CYP3A4* | CYP3A4 14269G>A | rs4986907 | 100 | 0.0 |
| *CYP3A4* | CYP3A4 14304G>A | rs4986908 | 100 | 0.0 |
| *CYP3A4* | CYP3A4 15603C>G | rs12721627 | 100 | 0.0 |
| *CYP3A4* | CYP3A4 15615T>C | rs4987161 | 100 | 0.0 |
| *CYP3A4* | CYP3A4 15626A>G | rs3208361 | 98.9 | 0.0 |
| *CYP3A4* | CYP3A4 15702C>G | rs55901263 | 100 | 0.5 |
| *CYP3A4* | CYP3A4 15713T>C | rs55785340 | 100 | 0.0 |
| *CYP3A4* | CYP3A4 16898T>G | rs3208363 | 100 | 0.0 |
| *CYP3A4* | CYP3A4 17661_176622insA | rs4646438 | 100 | 0.0 |
| *CYP3A4* | CYP3A4 20070T>C | rs28371759 | 98.9 | 1.1 |
| *CYP3A4* | CYP3A4 20230G>A | rs2242480 | 100 | 21.2 |
| *CYP3A4* | CYP3A4 21867C>T | rs67784355 | 100 | 0.0 |
| *CYP3A4* | CYP3A4 21896C>T | rs12721629 | 95.7 | 0.0 |
| *CYP3A4* | CYP3A4 22026C>T | rs4986909 | 100 | 0.0 |
| *CYP3A4* | CYP3A4 23127T>C | rs4986910 | 100 | 0.0 |
| *CYP3A4* | CYP3A4 23130T>C | rs1041988 | 98.9 | 0.0 |
| *CYP3A4* | CYP3A4 23231_23232insC | rs72552795 | 100 | 0.0 |
| *CYP3A4* | CYP3A4 23237C>T | rs4986913 | 100 | 0.0 |
| *CYP3A4* | CYP3A4 25889_25890insA | rs67666821 | 100 | 0.0 |
| *CYP3A5* | CYP3A5 3699C>T | rs55817950 | 100 | 0.0 |
| *CYP3A5* | CYP3A5 3705C>T | rs28383468 | 100 | 0.0 |
| *CYP3A5* | CYP3A5 3775A>G | rs72552791 | 100 | 0.0 |
| *CYP3A5* | CYP3A5 6986A>G | rs776746 | 100 | 26.6 |
| *CYP3A5* | CYP3A5 7249T>G | rs56244447 | 92.4 | 0.0 |
| *CYP3A5* | CYP3A5 7303C>A | rs41279857 | 97.8 | 0.0 |
| *CYP3A5* | CYP3A5 12952T>C | rs55965422 | 100 | 0.5 |
| *CYP3A5* | CYP3A5 14665A>G | rs56411402 | 100 | 0.0 |
| *CYP3A5* | CYP3A5 14690G>A | rs10264272 | 100 | 0.0 |
| *CYP3A5* | CYP3A5 19386G>A | rs28383479 | 100 | 0.0 |
| *CYP3A5* | CYP3A5 27131_27132insT | rs41303343 | 100 | 0.0 |
| *CYP3A5* | CYP3A5 27289C>A | rs28365083 | 93.5 | 0.0 |
| *CYP3A5* | CYP3A5 29753T>C | rs41279854 | 100 | 0.0 |
| *CYP3A5* | CYP3A5 31551T>C | rs28365085 | 100 | 0.0 |
| *FMO1* | FMO1 291C>G | rs56841822 | 98.9 | 0.0 |
| *FMO1* | FMO1 747C>T | rs742350 | 100 | 1.1 |
| *FMO1* | FMO1 907A>G | rs16864314 | 100 | 0.0 |
| *FMO1* | FMO1 1188A>G | rs1126692 | 100 | 1.1 |
| *FMO1* | FMO1 1504C>T | rs60639054 | 98.9 | 0.0 |
| *FMO1* | FMO1 *111C>T | rs12954 | 98.9 | 26.4 |
| *FMO1* | FMO1 *207C>T | rs7877 | 100 | 27.7 |
| *FMO3* | FMO3 94G>A | rs72549320 | 97.8 | 0.0 |
| *FMO3* | FMO3 154G>A | rs72549321 | 100 | 0.0 |
| *FMO3* | FMO3 182A>G | rs72549322 | 100 | 0.0 |
| *FMO3* | FMO3 198G>T | rs72549323 | 98.9 | 0.0 |
| *FMO3* | FMO3 245T>C | rs72549324 | 75.0 | 0.0 |
| *FMO3* | FMO3 394G>C | rs12072582 | 100 | 0.0 |
| *FMO3* | FMO3 441C>T | rs1800822 | 100 | 21.2 |
| *FMO3* | FMO3 442G>T | rs72549325 | 100 | 0.0 |
| *FMO3* | FMO3 458C>T | rs72549326 | 100 | 0.0 |
| *FMO3* | FMO3 472G>A | rs2266782 | 100 | 17.9 |
| *FMO3* | FMO3 596T>C | rs72549327 | 98.9 | 0.0 |
| *FMO3* | FMO3 604_605insT | rs72549328 | 71.7 | 0.0 |
| *FMO3* | FMO3 627+10C>G | rs2066534 | 100 | 1.1 |
| *FMO3* | FMO3 717T>C | rs1050902 | 100 | 0.0 |
| *FMO3* | FMO3 769G>A | rs1736557 | 96.7 | 14.0 |
| *FMO3* | FMO3 830T>C | rs2066530 | 76.1 | 0.0 |
| *FMO3* | FMO3 855C>T | rs909530 | 78.3 | 36.1 |
| *FMO3* | FMO3 906C>T | rs2066536 | 100 | 0.0 |
| *FMO3* | FMO3 913G>T | rs61753344 | 98.9 | 0.0 |
| *FMO3* | FMO3 923A>G | rs2266780 | 98.9 | 17.6 |
| *FMO3* | FMO3 940G>T | rs72549330 | 100 | 0.0 |
| *FMO3* | FMO3 1079T>C | rs28363581 | 100 | 0.0 |
| *FMO3* | FMO3 1084G>C | rs2066532 | 100 | 0.0 |
| *FMO3* | FMO3 1160G>T | rs72549331 | 60.9 | 0.0 |
| *FMO3* | FMO3 1458A>G | rs1050906 | 100 | 0.0 |
| *FMO3* | FMO3 1474C>T | rs72549334 | 98.9 | 0.0 |
| *FMO3* | FMO3 1507G>A | rs72549335 | 98.9 | 0.0 |
| *NR1I2* | NR1I2 -1135C>T | rs3814055 | 97.8 | 27.2 |
| *NR1I2* | NR1I2 34G>A | rs1063955 | 100 | 0.0 |
| *NR1I2* | NR1I2 79C>T | rs12721613 | 80.4 | 0.0 |
| *NR1I2* | NR1I2 106G>A | rs45610735 | 100 | 0.0 |
| *NR1I2* | NR1I2 292C>T | rs72551371 | 98.9 | 0.0 |
| *NR1I2* | NR1I2 365G>A | rs12721608 | 89.1 | 0.0 |
| *NR1I2* | NR1I2 418G>A | rs72551372 | 100 | 0.0 |
| *NR1I2* | NR1I2 443G>A | rs72551373 | 100 | 0.0 |
| *NR1I2* | NR1I2 488A>G | rs72551374 | 100 | 0.0 |
| *NR1I2* | NR1I2 684-93G>A | rs6785049 | 70.7 | 39.2 |
| *NR1I2* | NR1I2 768C>T | rs4058490 | 100 | 0.0 |
| *NR1I2* | NR1I2 827-17C>T | rs2276707 | 78.3 | 47.2 |
| *NR1I2* | NR1I2 997G>A | rs35761343 | 100 | 0.0 |
| *NR1I2* | NR1I2 1030C>T | rs72551375 | 95.7 | 0.0 |
| *NR1I2* | NR1I2 1096A>G | rs72551376 | 97.8 | 0.0 |
| *SULT1A1* | SULT1A1 404G>A | rs9282861 | 98.9 | 7.7 |
| *SULT1A1* | SULT1A1 433G>A | rs1801030 | 100 | 0.0 |
| *SULT1E1* | SULT1E1 -64G>A | rs3736599 | 100 | 28.8 |
| *SULT1E1* | SULT1E1 -9-899G>A | rs3775770 | 100 | 23.4 |
| *SULT1E1* | SULT1E1 -9-682A>G | rs4149528 | 98.9 | 7.7 |
| *SULT1E1* | SULT1E1 -9-469A>G | rs3822172 | 100 | 35.9 |
| *SULT1E1* | SULT1E1 -10+311G>C | rs1881668 | 100 | 35.9 |
| *SULT2A1* | SULT2A1 6G>A | rs11569685 | 100 | 0.0 |
| *SULT2A1* | SULT2A1 89T>C | rs17851828 | 100 | 0.0 |
| *SULT2A1* | SULT2A1 90C>T | rs11083907 | 98.9 | 0.0 |
| *SULT2A1* | SULT2A1 679A>G | rs11569680 | 100 | 0.0 |
| *SULT2A1* | SULT2A1 781G>A | rs11569679 | 97.8 | 0.0 |
| *SULT2A1* | SULT2A1 *161G>C | rs296365 | 80.4 | 33.8 |
| *UGT1A4* | UGT1A4 70C>A | rs6755571 | 95.7 | 0.0 |
| *UGT2B15* | UGT2B15 253G>T | rs1902023 | 100 | 48.9 |
| *UGT2B15* | UGT2B15 1498G>A | rs72551390 | 83.7 | 0.0 |
| *UGT2B15* | UGT2B15 1568A>C | rs4148269 | 100 | 14.7 |
| *UGT2B15* | UGT2B15 *131C>G | rs72551389 | 100 | 0.0 |
| *UGT2B15* | UGT2B15 *185A>T | rs4148271 | 100 | 22.8 |
| *UGT2B7* | UGT2B7 -327A>G | rs7662029 | 63.0 | 37.1 |
| *UGT2B7* | UGT2B7 -161T>C | rs7668258 | 98.9 | 35.2 |
| *UGT2B7* | UGT2B7 211G>T | rs12233719 | 97.8 | 11.7 |
| *UGT2B7* | UGT2B7 735A>G | rs28365062 | 97.8 | 2.2 |
| *UGT2B7* | UGT2B7 801A>T | rs7438284 | 91.3 | 32.7 |
| *UGT2B7* | UGT2B7 802T>C | rs7439366 | 88.0 | 27.8 |
| *UGT2B7* | UGT2B7 1062C>T | rs4348159 | 100 | 0.0 |

MAF, minor allele frequency.

Nucleotide numbering of *CYP1A2*, *CYP2B6*, *CYP2C19*, *CYP2C9*, *CYP2D6*, *CYP3A4*, *CYP3A5*, *UGT1A4*, *UGT2B15*, and *UGT2B7* genes was performed according to the homepages of the Human Cytochrome P450 (CYP) Allele Nomenclature Committee and UGT Alleles Nomenclature Committee.

Nucleotides were numbered according to accession number NM_002021.2 for *FMO1*, NM_006894.5 for *FMO3*, NM_033013.2 for *NR1I2*, NM_177536.3 for *SULT1A1*, NM_005420.2 for *SULT1E1*, and NM_003167.3 for *SULT2A1*.
